# EVERYTHING HAS CHANGED FOR THE WORSE: EXPERIENCES OF ELDERS DISPLACED BY ARMED CONFLICT IN ETHIOPIA

**DOI:** 10.1093/geroni/igac059.2131

**Published:** 2022-12-20

**Authors:** Getachew Gebeyaw, Shambel Dessale, Eyayu Kasseye, Margaret Adamek

**Affiliations:** University of Gondar, Gondar, Amhara, Ethiopia; University of Gondar, Gondar, Amhara, Ethiopia; University of Gondar, Gondar, Amhara, Ethiopia; Indiana University, Indianapolis, Indiana, United States

## Abstract

In November 2020 an outbreak of ethnically and politically motivated armed conflict started in Tigray, Ethiopia and expanded to the Amhara and Afar regions, bringing a devastating impact upon civilians and disadvantaged groups. Persons living in those areas, including elders, were forced to flee and seek refuge at internal displacement centers. The purpose of this study was to investigate the challenges faced by older individuals fleeing the war zone and settling in internal displacement centers during Ethiopia's armed conflict. A qualitative descriptive case study was used in this cross-sectional investigation. Purposive sampling was used to identify 13 displaced older adults. Data from the in-depth interviews with elders were supplemented with key informant interviews and observations. Narrative data was analyzed using thematic analysis. Findings revealed that the older adults faced a variety of challenges in the war zone while escaping their homes and in the displacement center, all of which negatively impacted their physical and psychosocial well-being. Elders fled on foot and walked for three days without food to reach the internal displacement center. In addition to food shortage, other themes included loss of significant others, family disintegration, and lack of care and support. The findings call attention to the need for practical access to social and economic integration of elders in the aftermath of war as well as ongoing psychosocial intervention. In Ethiopia and in other war-affected areas, displaced older people need tailored support.

