# Correction to: EVERYTHING HAS CHANGED FOR THE WORSE: EXPERIENCES OF ELDERS DISPLACED BY ARMED CONFLICT IN ETHIOPIA

**DOI:** 10.1093/geroni/igad024

**Published:** 2023-03-27

This is a correction to: Getachew Gebeyaw, Shambel Dessale, Eyayu Kasseye, Margaret Adamek, EVERYTHING HAS CHANGED FOR THE WORSE: EXPERIENCES OF ELDERS DISPLACED BY ARMED CONFLICT IN ETHIOPIA, Innovation in Aging, Volume 6, Issue Supplement_1, November 2022, Page 565, https://doi.org/10.1093/geroni/igac059.2131

In the originally published version of this abstract, affiliation 1 was erroneous and it has now been corrected to University of Gondar, Gondar, Amhara, Ethiopia.

